# Characterization and Comparison of 2 Distinct Epidemic Community-Associated Methicillin-Resistant *Staphylococcus aureus* Clones of ST59 Lineage

**DOI:** 10.1371/journal.pone.0063210

**Published:** 2013-09-05

**Authors:** Chih-Jung Chen, Clemens Unger, Wolfgang Hoffmann, Jodi A. Lindsay, Yhu-Chering Huang, Friedrich Götz

**Affiliations:** 1 Division of Pediatric Infectious Diseases, Department of Pediatrics, Chang Gung Memorial Hospital, Taoyuan, Taiwan; 2 College of Medicine, Chang Gung University, Taoyuan, Taiwan; 3 Institut für Tropenmedizin, Wilhelmstraße 27, Tübingen, Germany; 4 Department of Cellular & Molecular Medicine, St George’s, University of London, Cranmer Terrace, London, United Kingdom; 5 Department of Microbial Genetics, Faculty of Biology, University of Tübingen, Tübingen, Germany; Columbia University, United States of America

## Abstract

Sequence type (ST) 59 is an epidemic lineage of community-associated (CA) methicillin-resistant *Staphylococcus aureus* (MRSA) isolates. Taiwanese CA-MRSA isolates belong to ST59 and can be grouped into 2 distinct clones, a virulent Taiwan clone and a commensal Asian-Pacific clone. The Taiwan clone carries the Panton–Valentine leukocidin (PVL) genes and the staphylococcal chromosomal cassette *mec* (SCC*mec*) V_T_, and is frequently isolated from patients with severe disease. The Asian-Pacific clone is PVL-negative, carries SCC*mec* IV, and a frequent colonizer of healthy children. Isolates of both clones were characterized by their ability to adhere to respiratory A549 cells, cytotoxicity to human neutrophils, and nasal colonization of a murine and murine sepsis models. Genome variation was determined by polymerase chain reaction of selected virulence factors and by multi-strain whole genome microarray. Additionally, the expression of selected factors was compared between the 2 clones. The Taiwan clone showed a much higher cytotoxicity to the human neutrophils and caused more severe septic infections with a high mortality rate in the murine model. The clones were indistinguishable in their adhesion to A549 cells and persistence of murine nasal colonization. The microarray data revealed that the Taiwan clone had lost the ø3-prophage that integrates into the β-hemolysin gene and includes staphylokinase- and enterotoxin P-encoding genes, but had retained the genes for human immune evasion, *scn* and *chps*. Production of the virulence factors did not differ significantly in the 2 clonal groups, although more α-toxin was expressed in Taiwan clone isolates from pneumonia patients. In conclusion, the Taiwan CA-MRSA clone was distinguished by enhanced virulence in both humans and an animal infection model. The evolutionary acquisition of PVL, the higher expression of α-toxin, and possibly the loss of a large portion of the β-hemolysin-converting prophage likely contribute to its higher pathogenic potential than the Asian-Pacific clone.

## Introduction

Community-associated methicillin-resistant *Staphylococcus aureus* (CA-MRSA) strains are very successful pathogens that emerged in the late 1990s and spread throughout the world within a few years [1], [2]. The strains can be isolated from the skin and mucosa of a substantial proportion of healthy individuals and are capable of causing disease, including lethal infections [3]–[5]. Currently, CA-MRSA strains have outnumbered methicillin-susceptible *S. aureus* strains as the dominant community pathogen in many areas of the world [6], [7]. Epidemic CA-MRSA clones vary in different continents, countries, and even areas. For example, pulsed-field type USA300 (sequence type 8, ST8) and USA400 (ST1) are the major clones in the United States and Canada [8], [9]; ST80 clones are prevalent in Europe [10]–[12]; ST59 clones circulate in the Asian-Pacific area, including Taiwan and Australia; and ST30 clones are found worldwide, including the USA, Europe, Oceania, and Japan [13]–[17]. These 5 clones account for the majority of CA-MRSA infections worldwide. It is essential to elucidate the determinants contributing to the transmission and/or virulence of epidemic CA-MRSA clones.

Our previous molecular epidemiology studies in Taiwan on clinical and carriage isolates of CA-MRSA revealed that 2 major genotypes accounted for the majority of CA-MRSA strains [7], [18]. These were designated as a “Taiwan” clone and an “Asian-Pacific” clone, and were differentiated by pulsed-field typing. In addition, isolates of the Taiwan clone carried the type V_T_ SCC*mec* element and the Panton–Valentine leukocidin (PVL) genes, while isolates of the Asian-Pacific clone typically carried a type IV SCC*mec* element and lacked the PVL genes [17]. Isolates of both pulsotypes had a similar genetic background and belonged to the ST59 lineage. Severe infections were caused by the Taiwan clone (ST59-MRSA-V_T_-PVL-positive), although the Asian-Pacific clone (ST59-MRSA-IV-PVL-negative) was more prevalent in colonizing healthy individuals. In a carriage surveillance study of MRSA in 2001–2002, 78.3% of colonizing isolates belonged to the Asian-Pacific clone [19]. Another MRSA nasal carriage surveillance study in healthy Taiwanese children from 2005–2006 revealed that 62% of isolates belonged to the Asian-Pacific clone and 28% to the Taiwan clone [20]. In contrast, 73% of CA-MRSA infections in Taiwanese children were caused by the Taiwan clone in a prospective study during 2004–2005 [7]. Strains carrying type V_T_ SCC*mec* elements (presumed to be the Taiwan clone) constituted 71.1% of clinical isolates of MRSA with CA characteristics in an additional island-wide survey [21].

The above epidemiological observations suggest a greater virulence for the Taiwan clone as compared to the Asian-Pacific clone. The respective abundance of the 2 related clones in colonizing strains and infecting strains provided an excellent opportunity to explore the factors implicated in CA-MRSA pathogenesis. To achieve this goal, we first compared the phenotypes of the 2 clones, including the ability to colonize and infect, by using murine models. Second, the genetic compositions of the 2 clones were delineated by polymerase chain reaction (PCR) screening of selected virulence genes and by comparative genomics using a DNA microarray. The genomic study also helped to elucidate the evolutionary history of the ST59 strains. Finally, the expression of virulence genes most likely to be contributing factors to the pathogenesis of CA-MRSA was analyzed and compared between the 2 clones.

## Results

### Adhesive Assay and Murine Colonization Study

**Figure 1:**
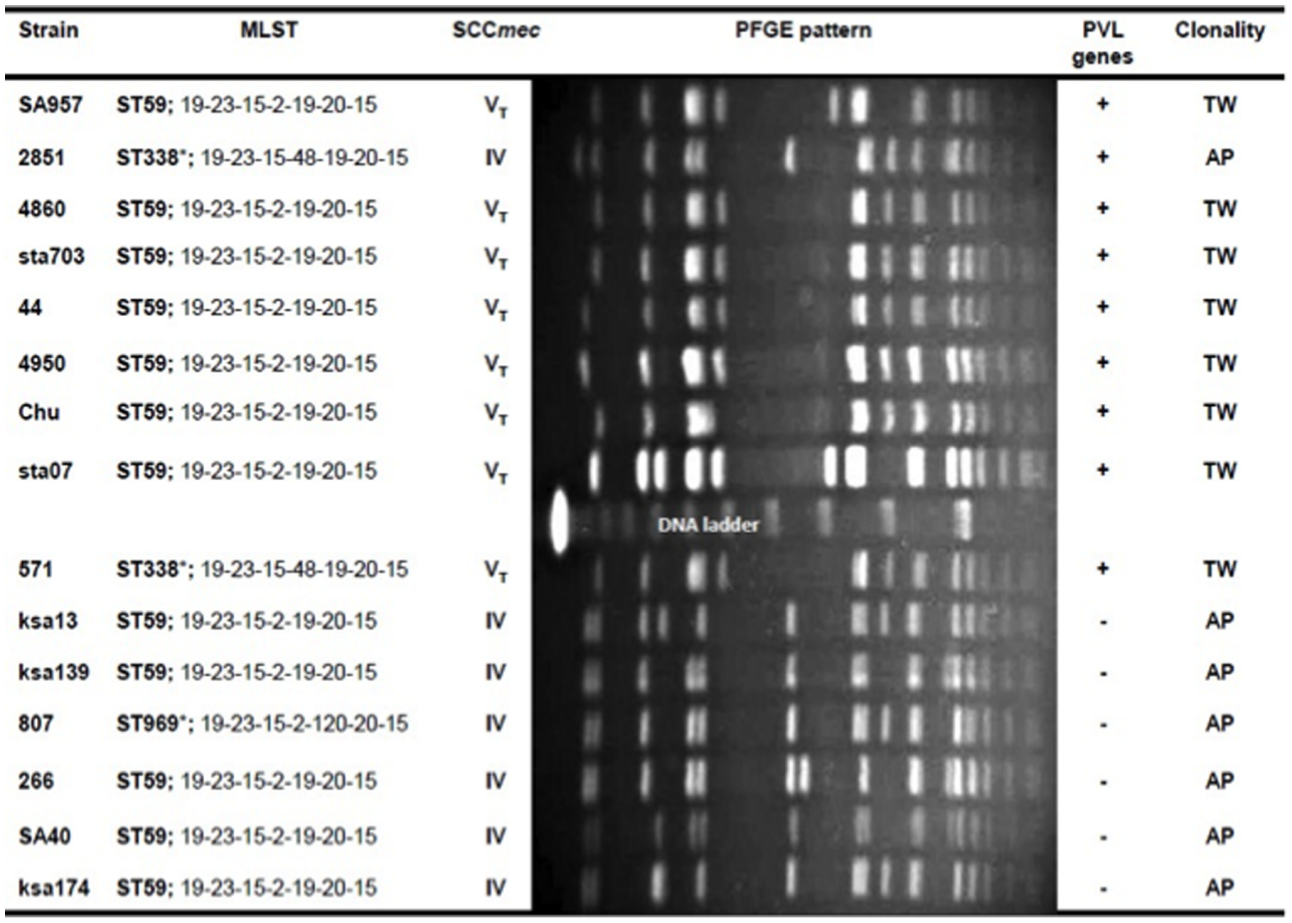
Genotypes of 15 CA-MRSA strains used in the current study. *ST338 is a single locus variant of ST59 Abbreviations: MLST, multi-locus sequence type; SCC*mec*, staphylococcal chromosomal cassette *mec*; PFGE, pulsed-field gel electrophoresis; PVL, Panton–Valentine leukocidin; TW, Taiwan clone; AP, Asian-Pacific clone.

A comparison of 15 CA-MRSA isolates of ST59 lineage (Table 1 and Figure 1) belonging either to the Asian-Pacific clone or Taiwan clone revealed no differences in growth rate or biofilm production (Figure S1a–c). Susceptibility to various non-β-lactam antibiotics was also similar for the 2 clones, although tetracycline was an exception because the Taiwan clones were more frequently resistant to it than the Asian-Pacific clones (100% vs. 42.9%, *p* = 0.026; Figure S1d).

**Table 1:** Characteristics of CA-MRSA strains used in the current study.

Strain name	Characteristics of isolates			Characteristics of host			Reference
	Source	Origin	Year	Age (y)/sex	Pneumonia	Other clinical symptoms	
SA957	Blood	NT	2000	16.4/Male	+	Bacteremia, arthritis, cutaneous abscess	[4]
2851	Abscess	NT	2001	16.1/Male	−	Cutaneous abscess, shock	[4]
4860	Blood	NT	2001	13.3/Male	+	Bacteremia, arthritis	[4]
sta703	Blood	NT	2007	11.8/Male	+	Bacteremia, pyomyositis, deep venous thrombosis, ICH	Current study
44	Blood	NT	2004	11.2/Female	+	Bacteremia, arthritis, osteomyelitis, pyomyositis	[4]
4950	Pleural fluid	NT	2001	0.1/Male	+	Pleural empyema	[4]
chu	Blood	NT	2003	12.4/Male	+	Bacteremia, osteomyelitis, arthritis	[4]
sta07	Abscess	NT	2004	0.6/Male	−	Cutaneous abscess	Current study
571	NP swab	CT	2006	4.4/Female	−	Healthy child	[3]
ksa13	NP swab	ST	2006	NA	−	Healthy child	[3]
ksa139	NP swab	ST	2005	NA	−	Healthy child	[3]
807	NP swab	CT	2006	2.3/Female	−	Healthy child	[3]
266	NP swab	CT	2005	4.4/Female	−	Healthy child	[3]
SA40	NP swab	NT	2005	NA	−	Healthy child	[3]
ksa174	NP swab	ST	2006	NA	−	Healthy child	[3]

Abbreviations: CT, central Taiwan; NA, not available; NP, nasopharyngeal; NT, northern Taiwan; ST, southern Taiwan.

**Figure 2:**
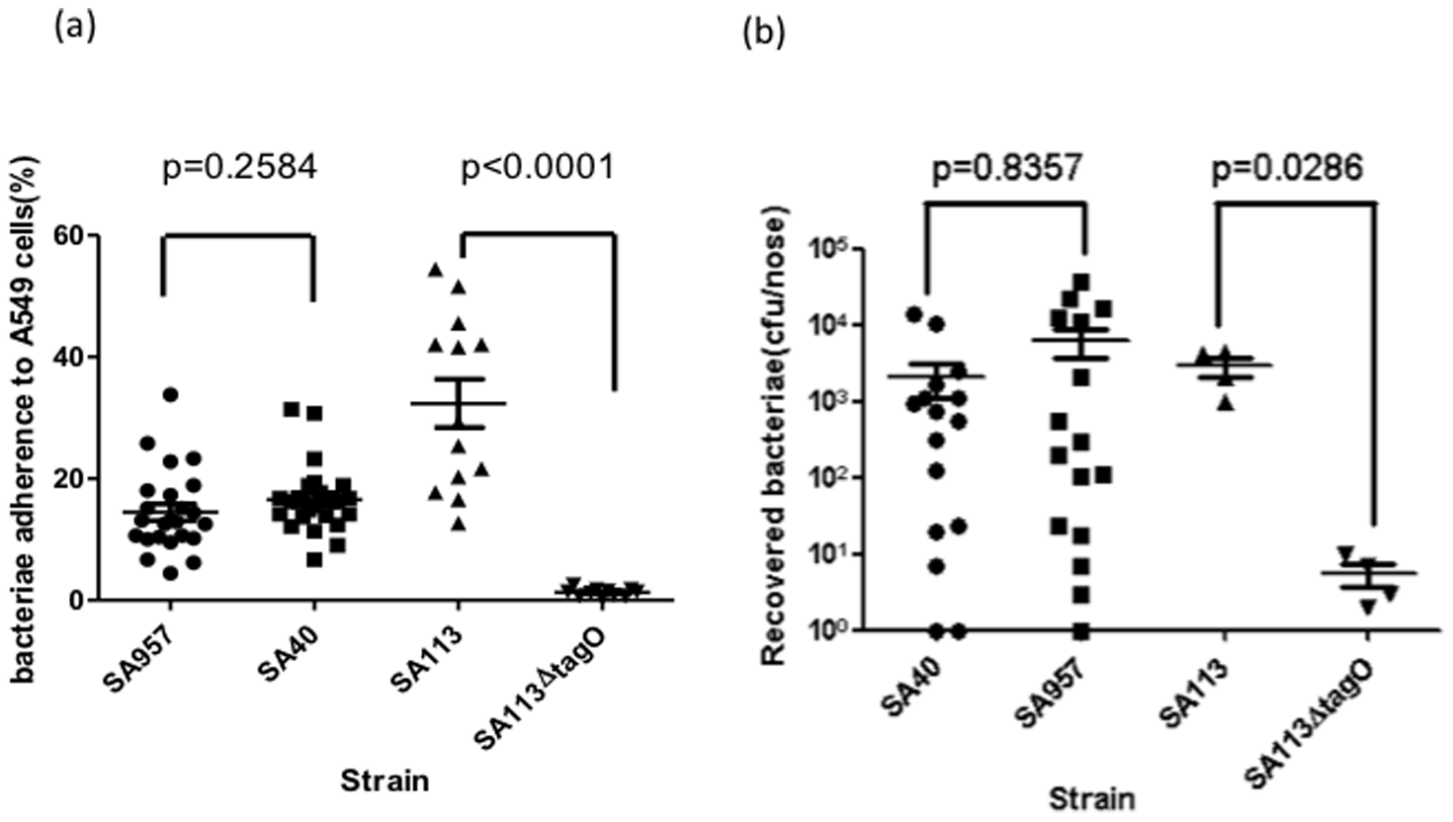
Comparisons of adherence to respiratory epithelial cells (a) and colonization in murine nares (b) in 2 representative strains of the Taiwan clone (SA957) and the Asian-Pacific clone (SA40) of CA-MRSA ST59. The adherence experiment was repeated 6 times for SA957 and SA40 and repeated 3 times for SA113 and SA113Δ*tagO*. Each strain was replicated 4 times in each experiment. Significance of differences was determined by two-way ANOVA. For murine nasal colonization, SA957 and SA40 were tested in 16 mice; SA113 and SA113 Δ*tagO* were tested in 4 mice. Significance was determined by Mann–Whitney test.

The predominance of the Asian-Pacific clone in CA-MRSA carriage may be due to the higher colonizing ability of this clone than the Taiwan clone. To test this hypothesis, adherence to respiratory epithelium cells (A549) was determined and compared between 2 representative strains (SA40 and SA957) belonging to the Asian-Pacific clone and Taiwan clone, respectively. The assay was validated by showing a significant defect in adherence using the SA113 strain containing a *tagO*-deletion, a factor essential for nasal colonization (Figure 2a). The adherence to A549 cells was similar for both SA40 and SA957. Colonization ability was further tested *in vivo* in a murine nasal carriage model. After 7 days of instillation, the colony counts of bacteria recovered from the nose of mice were not significantly different between SA40 and SA957 (Figure 2b). These results failed to support the hypothesis that greater colonization ability was responsible for the abundance of the Asian-Pacific clone in CA-MRSA carriage isolates. However, these colonization assays were carried out using a human alveolar basal epithelial cell line and a murine nasal carriage model, which might not be equivalent to human nasal carriage.

### Cytotoxicity Assay and Infectivity in Murine Sepsis Model

**Figure 3:**
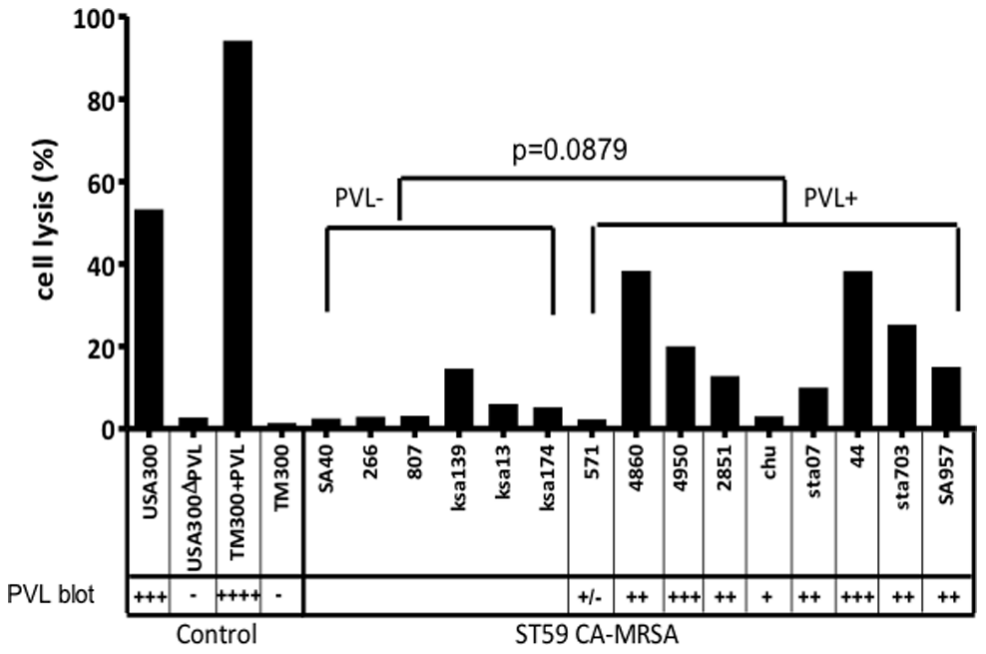
Cytotoxic effect of an overnight bacterial culture supernatant on human neutrophils. The cytotoxicity was compared between PVL-positive strains and PVL-negative strains of CA-MRSA of ST59 lineage.

To evaluate the virulence of the Asian-Pacific clone and Taiwan clone, the cytotoxicity of an overnight culture supernatant to human PMN cells was determined using 15 ST59 CA-MRSA isolates (Table 1 and Figure 1). Isolates carrying the PVL genes (mainly the Taiwan clone) had a higher cytotoxicity to human PMN cells than did isolates of the PVL-negative Asian-Pacific clone, although this did not reach statistical significance (*p* = 0.0879; Figure 3). The cytotoxic effect on neutrophils correlated well with the amount of PVL in the culture supernatant (Figure 3).

**Figure 4:**
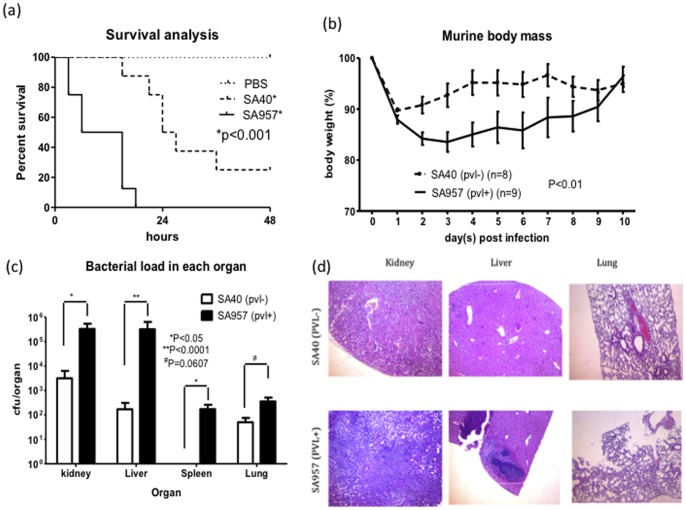
Comparison of Taiwan clone and Asian-Pacific clone virulence in a murine bloodstream infection model. Isolates of the Taiwan clone (SA957) were more lethal at a lethal dosage (5×10^7^ CFU) (a), caused greater body weight loss (b), and greater bacterial loads in various organs (c) at a non-lethal dosage (5×10^6^ CFU) than isolates of the Asian-Pacific clone (SA40). More prominent neutrophil aggregation was also seen upon histologic examination of liver, kidney, and lung of mice infected with SA957 at a lethal dosage (d). Survival analysis was performed using a Log-rank (Mantel–Cox) test (a). Comparisons of body weight changes of mice (b) and the bacterial loads in each organ (c) were performed using two-way ANOVA and Mann–Whitney tests, respectively.

To further evaluate the virulence of the 2 epidemic representatives, SA40 (Asian-Pacific clone) and SA957 (Taiwan clone) were injected into the bloodstream of 2 groups of mice treated with either a lethal (5×10^7^ colony-forming units, CFU) or with a non-lethal (5×10^6^ CFU) dosage of bacteria. Mortality after 48 h was not significantly different for mice infected with lethal dosages of SA957 (100%) and SA40 (75%, *p* = 0.467; Figure 4). However, all deaths in the SA957-infected mice occurred within 24 h of infection. Mice infected with SA40 had a longer survival time as shown by Kaplan–Meier survival analysis (*p*<0.001; Figure 4a). A greater virulence of SA957 was also indicated by significantly more body weight loss and bacterial loads in various internal organs of mice infected with non-lethal dosages of SA957 than with SA40 (Figure 4b–4d).

### Comparative Genomics

**Figure 5:**
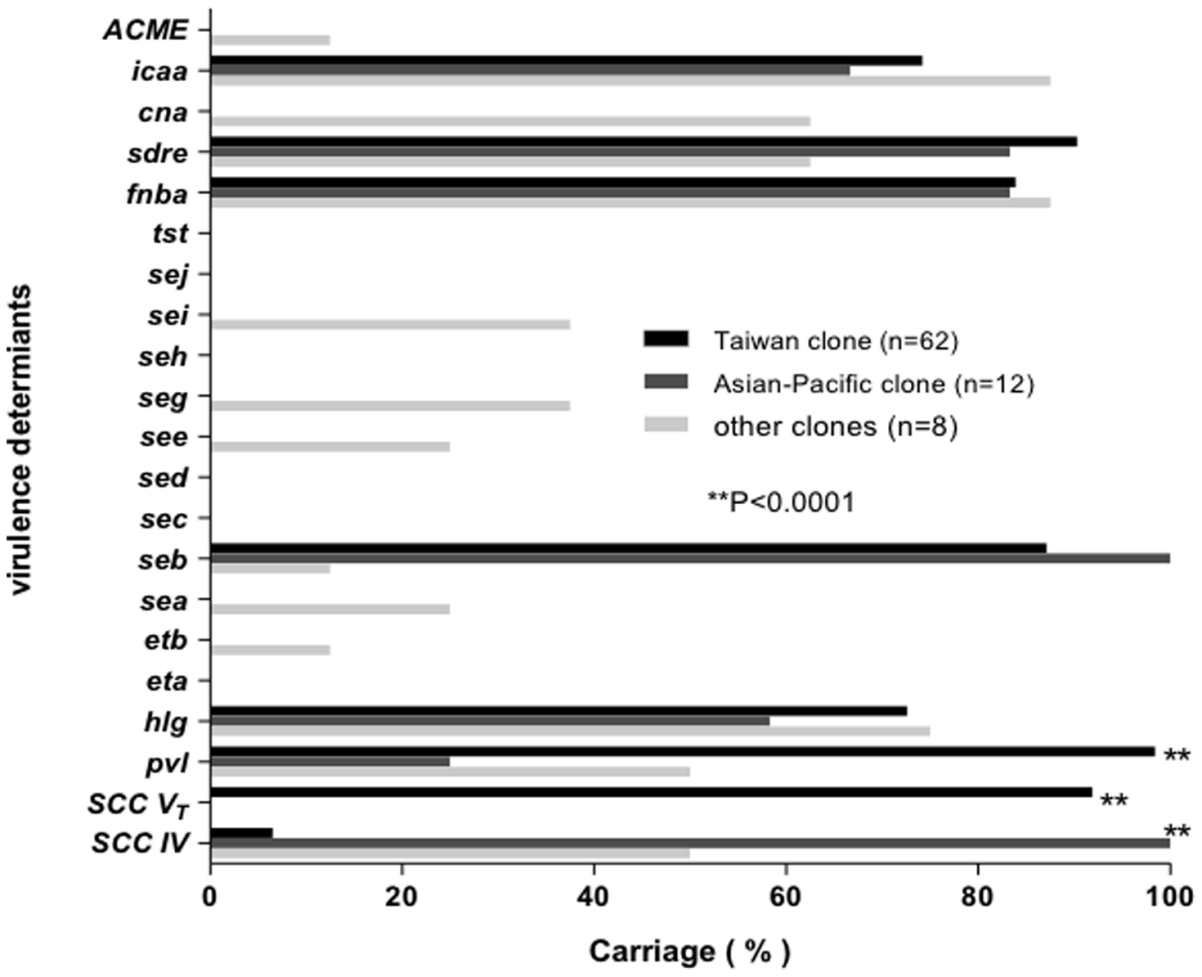
Distribution of selected virulence determinants in 82 random samples of clinical community-associated MRSA isolates from Taiwanese children. The carriage rates of virulence determinants are compared between Taiwan clones and Asian-Pacific clones.

To further explore the determinants of virulence of the 2 epidemic CA-MRSA clones, we determined pulsotypes in a random sample of 82 clinical CA-MRSA strains from Taiwanese children during 2003–2007. The strains were further screened for the presence of genes encoding 10 superantigens (SEA-SEE, TSST-1, and SEG-SEJ), 2 exofiliatin toxins (ETA and ETB), 2 cytolysins (PVL and γ-hemolysin), 4 adhesion proteins (FnbA, SdrE, cna and icaA), arginine catabolic mobile elements (ACME), and SCC*mec* types by PCR (Figure 5). As expected, there was a strong association between clonality (pulsotypes) and SCC*mec* types. The majority (91.9% of 62 isolates) of Taiwan clones carried type V_T_ SCC*mec* whereas 12 (100%) isolates of the Asian-Pacific clone carried type IV SCC*mec* (*p*<0.0001, Fisher’s exact test). All isolates of both the Taiwan clone and the Asian-Pacific clone were negative for cna and ACME genes as well as most of the screened cytotoxins with the exception of enterotoxin B, PVL, and γ-hemolysin. Enterotoxin B and γ-hemolysin were identified in 66 (89.2%) and 52 (70.3%) isolates, respectively. The presence of enterotoxin B, γ-hemolysin, and genes encoding adhesion proteins such as FnbA, SdrE, and icaA did not differ significantly between the 2 epidemic clones of ST59. PVL genes were harbored by 64 (86.5%) of 82 isolates and were identified significantly more often in the Taiwan clones (98.4%) than in the Asian-Pacific clones (25.0%, *p*<0.0001).

**Figure 6:**
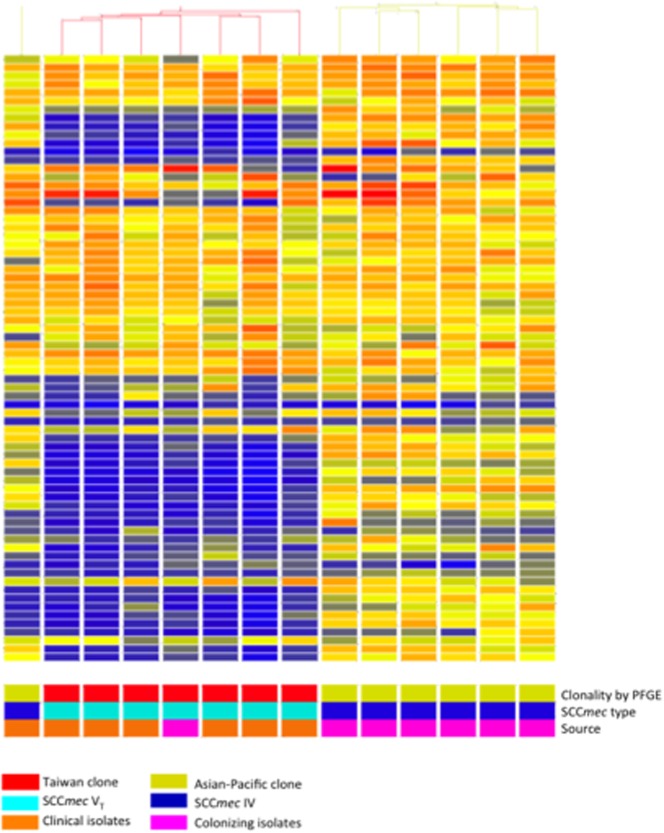
Microarray hybridization data for 14 isolates of CA-MRSA visualized using phage Sa3 genomes of MRSA 252. Relatedness between the isolates is illustrated by the dendrogram. The isolates were grouped into 2 clusters, which is consistent with the pulsed typing grouping result. Each of the color blocks represents a PCR product and is colored according to the normalized ratio, with color intensity dependent on the signal for MRSA252 by using GeneSpring software. The bright colors (i.e., red, yellow, and orange) indicate that the genes are present, whereas the dark colors (i.e., blue and gray) indicate that the genes are absent or divergent. The data suggest that the isolates of PVL-positive Taiwan clone carry a truncated Sa3 prophage in which 2 DNA fragments are missing.

To obtain more information of the genetic compositions of the 2 epidemic ST59 clones, we conducted comparative genomics with 14 CA-MRSA strains of ST59 lineage (Table 1 and Figure 1) by using a microarray containing 3,626 open reading frames (ORFs). The array data can be accessed in ArrayExpress (accession number E-MEXP-3627). The microarray analysis clustered the 14 CA-MRSA strains into 2 groups, consistent with the grouping of the pulsed-field pattern. Among the 3,626 ORFs in the gene chip, 2,984 (82.3%) were present in at least 6 of 7 Taiwan clone isolates and 2,840 (78.3%) were present in at least 6 of 7 Asian-Pacific clone isolates. Three ORFs, encoding hypothetical proteins and phage integrase, were specific to the Taiwan clone (Table S1). Twenty-eight ORFs were specific for colonizing strains (Table S1), which included 22 genes on a β-hemolysin-converting prophage (øSa3 in MRSA252), 5 SCC*mec*-associated genes, and 1 gene of unknown function on core genome. The microarray hybridization data for the 14 strains of CA-MRSA were visualized using the bacteriophage øSa3 genome in MRSA252 (Figure 6). It showed that most of the genes in øSa3 were present in isolates of the Asian-Pacific clone, but 2 segments (dark-colored blocks) were missing in the isolates of the Taiwan clone. The missing segment included genes such as *sak* (encoding staphylokinase) and *sep* (encoding enterotoxin P).

### Expression of Selected Global Regulators and Virulence Factors

**Figure 7:**
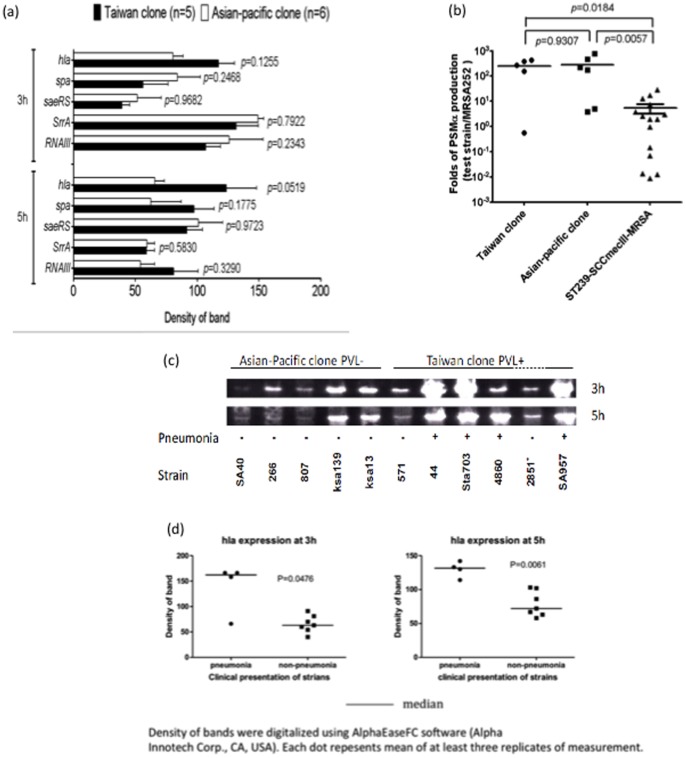
Expression of global regulators and virulence factors. Northern blot analysis did not show significant differences in the expressions of *RNAIII, SrrA, seaRS, spa,* and *hla* for isolates of the Taiwan and Asian-Pacific clones (a). The production of PSM-α as measured by qPCR was also similar for isolates of the 2 clones. The PSM-α production in a prevalent nosocomial MRSA clone in Taiwan, ST239 with type III SCC*mec,* is also displayed, which is significantly less than the production in the 2 clones of ST59 lineage. (b). The expression of α-toxin was significantly higher in isolates causing pneumonia than nasal isolates and isolates causing diseases other than pneumonia (c, d). The data represent 3 replicates for each virulence factor (d).

The expression of *RNAIII*, *srrA*, and *saeRS*, as well as 3 important virulence factors (α-hemolysin, phenol-soluble modulins-α (PSM-α), and protein A) were compared between 11 isolates of the 2 epidemic clones, which included 5 Taiwan clone isolates and 6 Asian-Pacific clone isolates (Figure 7c). The expression of protein A, PSM-α, and global regulators, including *RNAIII*, *SrrA*, and *saeRS*, was not significantly different between the 2 clones (Figure 7a–b). The α-toxin was produced at higher levels in 4 of the 5 Taiwan clone strains than those of the Asian-Pacific clone, but this did not reach statistical significance (Figure 7a and 7c). Interestingly, all 4 strains producing high levels of α-toxin were PVL-positive and were isolated from children with severe CA-MRSA pneumonia. The other 2 PVL-positive strains with low levels of α-toxin production were either a nasal isolate (strain 571) or a wound culture isolate (strain 2851) (Figure 7c).

## Discussion

Nasal colonization of *S. aureus* is a known risk factor for the development of subsequent *S. aureus* diseases in selected populations. Data from previous epidemiology studies have suggested that carriage of *S. aureus* strains with certain characteristics (e.g., harboring PVL genes, methicillin resistance) was associated with increased risk of subsequent infections [22], [23]. The epidemic surveillance data of CA-MRSA in Taiwan was in agreement with previous observations and strongly suggests that a PVL-positive clone, i.e., the Taiwan clone, had a greater potential of causing severe disease as compared to its sister clone, the PVL-negative Asian-Pacific clone. The epidemiologic observation was supported by the results of the current study, which showed that there was an increased pathogenic potential for the Taiwan clone in both an *in vitro* human PMN cytotoxicity assay and an *in vivo* murine sepsis model.

We were not successful in identifying molecular determinants that would account for the increased virulence of the Taiwan clone in microarray study. The genome-wide comparison disclosed only very subtle differences in genetic compositions between the 2 clones. The Taiwan clone did not harbor any extra known virulence determinants, with the exception of PVL genes, in comparison to the Asian-Pacific clone. Given the inherent limitations of the DNA microarray, some virulence factors may not have been readily identified. However, we believe that this is unlikely due to preliminary results of whole genome sequencing of the 2 representative strains, SA957 (Taiwan clone, accession number CP003603, GenBank) and SA40 (Asian-Pacific clone, accession number CP003604, GenBank) showing very similar findings to that of the microarray data (Prof. Cheng-Hsun Chiu, unpublished data). The failure to identify Taiwan clone-specific virulent determinants other than PVL might suggest a potential role of PVL in the pathogenic success of the Taiwan clone. Nevertheless, small genetic variations such as single nucleotide polymorphisms resulting in non-synonymous mutations are very difficult, if not impossible, to be captured by DNA microarray analysis. Single nucleotide alterations can have a major impact on the function of the affected alleles and may be contributing to the different virulence profile of the 2 strains. A comprehensive comparison of the 2 genomes at the nucleotide level is necessary to address this issue, and is currently being undertaken.

The comparative genomic data suggest that, before acquiring the PVL prophage and methicillin resistance, the progenitor of the Taiwan clone might have undergone at least 2 recombination episodes, ultimately resulting in the loss of 2 DNA fragments from the Hlb-converting prophage, øSa3 [17], [24]. These fragments contained genes encoding immune evasion molecules such as staphylokinase and enterotoxin P. Approximately 90% of *S. aureus* strains carry the immune evasion cluster (IEC) with varying combinations of *scn* (staphylococcal complement inhibitor), *chp* (chemotaxis inhibitory protein), *sea* or *sep*, and *sak* [25]. The partial loss of the IEC in the more virulent Taiwan clone suggests that the molecules of IEC (e.g., *sak* and *sep*) do not contribute to the high virulence of the Taiwan clone. Instead, loss or truncation of the “virulence genes” (e.g., *sak*) during evolution may increase bacterial fitness in hosts, thus conferring a more virulent phenotype [26]. The association of genome reduction with increased virulence has been documented in comparative studies utilizing a variety of epidemic bacteria [27]–[29]. Indeed, this hypothesis was supported by the observation that there was a higher frequency and a greater magnitude of staphylokinase production in staphylococcal isolates from nasal swabs than from bloodstreams [30]. A clinical study further showed that *sak*-deficient *S. aureus* strains were associated with significantly worse clinical outcomes in patients with bacteremia compared to patients infected with isolates producing high levels of staphylokinase [31]. These observations together also suggest that the IEC molecules might contribute to the predominance of the Asian-Pacific clone in the nasal MRSA isolates. Unfortunately, the *in vitro* assay of adherence to respiratory cells and a murine nasal carriage model failed to demonstrate a greater adherence and colonization ability of the Asian-Pacific clone compared to the Taiwan clone. In an animal model, this may be explained by murine non-susceptibility to the IEC molecules [32], [33]. Alternatively, the higher frequency of the Asian-Pacific clone in the carriage CA-MRSA isolates may be because of a greater expression of some surface proteins, which were not thoroughly characterized in this study and further studies will be warranted to confirm this speculation.

Human neutrophils represent the first line of defense against bacterial invasion. *S. aureus* excretes a variety of exotoxins that are cytotoxic to many types of host cells, including human neutrophils [34]. PVL has been shown to be a potent cytotoxic factor for human neutrophils [35]; however, the role of PVL in the pathogenesis of CA-MRSA infection has been an issue of debate due to controversial data generated by different study groups [36]–[38]. Our data indicate that the supernatant of the Taiwan clone induced a greater degree of neutrophil lysis than the Asian-Pacific clone. Furthermore, the extent of neutrophil lysis appeared to be correlated with the production of PVL in the Taiwan clone, suggesting that PVL at least in part accounted for the cytotoxicity to neutrophils in the ST59 CA-MRSA strains. PSM is a group of novel cytolytic peptides of *S. aureus*. In addition to the cytotoxicity to human neutrophils, the newly identified cytolysins have recently been shown to have proinflammatory activity and are considered essential virulence factors of CA-MRSA strains [39]. Indeed, PSM-α, the most potent PSM cytolysin, showed significantly higher expression in ST59 CA-MRSA strains than in the nosocomial MRSA strains of a pandemic clone, ST239 (Figure 7b). However, the expression of PSM-α was not significantly different in the Taiwan and Asian-Pacific clones, suggesting that PSM-α plays only a minor role in human neutrophil cytotoxicity for the CA-MRSA ST59 strains, and that it does not account for the high pathogenicity of the Taiwan clone.

α-toxin is able to destroy a variety of host cells, including respiratory epithelial cells, and has been shown to be essential for the pathogenesis of staphylococcal pneumonia in mice [40]. The significance of α-toxin in staphylococcal pneumonia was further supported by the finding that immunization of mice against α-toxin can protect them from *S. aureus* pneumonia [41]. The high abundance of α-toxin production was also considered an important factor for the enhanced virulence of the epidemic CA-MRSA clone, USA300 [42]. Our data further indicates that, compared to isolates from other sources, the expression of α-toxin was significantly higher in CA-MRSA isolates causing pneumonia.

Intriguingly, all pneumonia isolates also harbored the PVL genes. The significant association of PVL gene carriage and *S. aureus* pneumonia has been noted in previous observations by our group and others [4], [43], [44]. The role of PVL in staphylococcal pneumonia was also shown in a study using a rabbit model of necrotizing pneumonia [45]. Purified PVL was sufficient to recruit and lyse PMN cells and damage lung tissue. Taken together, these data suggest an important role of α-toxin and PVL in the pathogenesis of CA-MRSA pneumonia.

In conclusion, comparison of 2 related epidemic CA-MRSA clones of ST59 lineage indicated that the PVL-positive Taiwan clone was a much more virulent and disease-prone clone compared to the PVL-negative Asian-Pacific clone. The presence of PVL and the higher expression of certain virulence molecules, the most important of which is α-toxin, most likely contribute to the high pathogenic potential of the Taiwan clone. Since PSM-α is equally produced in isolates of both clones, it does not account for the high virulence of the Taiwan clone. Comparative genomic data suggest that the β-hemolysin-converting bacteriophage (øSa3) genome was partially truncated in the Taiwan clone, but was intact in the Asian-Pacific clone. This convergent evolution resulting in gene loss and acquisition may contribute to the success of the epidemic CA-MRSA clone in the past decade.

## Methods

### Ethics Statement

All animal experiments were approved by the ethics committee of Chang Gung Memorial Hospital. Animal care and use was in accordance with the institutional guidelines set forth by Chang Gung Memorial Hospital. The cytotoxicity study of human neutrophil was conducted in Münster University. Human blood sample acquisition and cell isolations were conducted with the approval of the local ethics committee (Ethik-Komission der Ärztekammer Westfalen-Lippe und der Medizinischen Fakultät der Westfälischen Wilhelms-Universität Münster). Human blood samples were obtained from healthy blood donors, who provided written informed consent for the collection of samples and subsequent neutrophil isolation and analysis.

### Bacterial Strains and Growth Condition

Fifteen MRSA strains with previously determined genotypes were used in the current study. Of these, 8 strains were isolated from hospitalized children with various clinical syndromes between 2000 and 2007. We arbitrarily used strains with different clinical manifestations, including cutaneous abscess, bone/joint infections, pneumonia, deep venous thrombosis, pyomyositis, bacteremia, and shock (Table 1). The clinical strain selection was not random because we intended to explore if a specific disease entity was associated with any characteristics of the strain (i.e., the harbored genes or the expression of particular factors). Seven strains were randomly selected nasal isolates from healthy children from a surveillance study between 2005 and 2008 [3]. The detailed clinical information and molecular types of the strains are shown in Table 1 and Figure 1. Two representative strains denoted the Taiwan clone (SA957) and Asian-Pacific clone (SA40), which had been chosen for a separate whole genome-sequencing project, were selected for characterization in the experiments of adherence to respiratory epithelial cells, colonization, and bloodstream infections in murine models. An additional set of 82 randomly selected clinical MRSA isolates from pediatric patients between 2003 and 2007 were screened for selected virulence factors by using PCR (Methods S1, Table S2). Bacteria were grown in liquid tryptic soy broth (TSB) (Sigma, Munich, Germany) or on solid BM (1% Peptone, 0.5% yeast extract, 0.5% NaCl, 0.1% K_2_HPO_4_, and 0.1% glucose) [46] unless otherwise noted. A wall teichoic acid-deficient mutant (Δ*tagO*) and its isogenic parent strain SA113 were included in the assay of bacterial adherence to A549 cells and murine nasal colonization model [47].

### Assay of Bacterial Adherence to A549 Cells

A549 cells (2×10^5^ in 2 mL DMEM medium per well) were prepared in 6-well plates and incubated overnight. The bacterial isolates were grown in 15 mL TSB medium to an OD_578_ of 0.5, washed, and resuspended in 15 mL PBS. Next, 100 µL of washed bacteria were inoculated onto the A549 cells and centrifuged at 500 rpm for 5 min to enhance adhesion. The same volume of bacteria was added to another plate with DMEM alone as a control. After 1 h of incubation at 37°C, the wells were washed 3 times with PBS, and the cells were detached by adding 150 µL of 0.25% trypsin. The cells were further lysed by addition of Triton-X100 at a concentration of 0.025%. The lysates were serially diluted and plated out for counting of bacterial numbers. SA113, SA113Δ*tagO*, and representative strains for the Taiwan clone (SA957) and the Asian-Pacific clone (SA40) were tested in this assay. Each strain was replicated 4 times in each experiment. The experiment was repeated 6 times for SA957 and SA40 and 3 times for SA113 and SA113 Δ*tagO*.

### Murine Nasal Colonization Study

The murine nasal colonization model was modified from a previously published protocol [48]. The overnight-cultured bacteria were diluted 1∶100 and grown by shaking at 150 rpm to an OD_578_ of 0.8 in TSB medium at 37°C. A predetermined volume of bacterial suspension equivalent to 1×10^8^ CFU was precipitated by centrifugation, washed with PBS, and then resuspended in 10 µL PBS. Six- to 8-week-old C57BL/6 mice were purchased from the National Laboratory Animal Center. Drinking water containing streptomycin (5 g per 1 L water) was provided for at least 48 h before inoculation and through the whole study. Next, 10 µL of a bacterial suspension containing 1×10^8^ CFU of bacteria was pipetted slowly onto the nares of the mice without actually touching the pipette tip to the nose. The animals were killed using CO_2_ and evaluated for nasal carriage of bacteria 7 d after inoculation. The external nasal region was wiped with 70% ethanol, excised from the inside front of mouth, dissected with sterile scissors, and vortexed vigorously in 1 mL PBS. The suspension was serially diluted and plated out for counting of bacterial numbers. Baird–Parker medium (BD-Taiwan, Becton, Dickinson and Company) was used for selection of *S. aureus* strains. SA957 and SA40 were tested in 16 mice. SA113 and SA113Δ*tagO* were tested in 4 mice.

### Murine Sepsis Model

The murine sepsis model was performed in accordance with the method described previously with some modifications. Briefly, 6- to 8-week-old C57BL/6 mice were injected with the indicated *S. aureus* strain at a lethal dosage of 5×10^7^ CFU or a non-lethal dosage of 5×10^6^ CFU via the jugular vein. At the lethal dosage, each group contained 8 mice. At the non-lethal dosage, 9 mice were infected with SA957 and 8 mice were infected with SA40. The mice infected with the lethal dosage were observed at 3 h intervals for symptoms of hunched posture, decreased activity, ruffled fur, and labored breathing. The mice were killed if they were too sick to eat or drink, or if they became immobile. All surviving mice were killed at 48 h. At the time of death, kidneys, liver, and lungs were removed and fixed in 10% neutral-buffered formalin. After dehydration using a graded series of ethanol, the tissues were embedded in paraffin, sectioned, and stained with hematoxylin and eosin. The mice infected with the non-lethal dosage were observed and weighed every 12 h for 7 d. All animals were killed at day 7, and the kidneys, liver, spleen, and lungs were removed for homogenization and determination of bacterial load.

### Preparation of Human Neutrophils and Cytotoxicity Assay

Human neutrophils were freshly isolated from Na citrate-treated blood of healthy adult volunteers. Dextran-sedimentation and density gradient centrifugation using Ficoll-Paque Plus (Amersham Bioscience) was used for neutrophil isolation, according to the manufacturer’s instruction. Neutrophils were resuspended at a final density of 1×10^6^ cells/0.5 mL in RPMI 1640 culture medium (PAA Laboratories GmbH) supplemented with 10% heat-inactivated FCS (PAA Laboratories GmbH). Neutrophils were used immediately for cytotoxicity assays.

All cytotoxicity experiments were performed in 24-well plates, and neutrophils were incubated with bacterial supernatants of overnight cultures grown in brain-heart infusion broth in a rotary shaker. Supernatants were sterile-filtered and added to the cell culture medium at a final concentration of 30%. All incubations were performed at 37°C in humidified air with 5% CO_2_. Measurement of cell death was performed after 1 h of incubation, followed by washing and staining of cells with propidium iodide. The cells were analyzed in a FACS caliber flow cytometer.

### PVL Blot

Detection of PVL (lukF and lukS) was performed following methods that were previously described [35]. Anti-PVL antibodies were raised in rabbits, followed by incubation with anti-rabbit alkaline phosphatase-conjugated antibodies, and bands were visualized colorimetrically using avidin alkaline phosphatase. To detect PVL amounts in bacterial culture supernatants, bacteria were grown in 5 mL of brain-heart infusion medium, supernatants were sterile-filtered, and used for western blot analysis. The amount of PVL was determined semi-quantitatively using 5 categories: −, no PVL production; +/−, borderline; +, low; ++, high; and +++, very high PVL production.

### Genomic Microarray

Comparative genomics was performed using a whole-genome *S. aureus* DNA microarray containing 3,623 probes, which was designated and validated by Witney et al. The 3,623 probes on microarray chip represented every predicted ORF of 7 whole-genome sequenced *S. aureus* strains (MRSA252, N315, Mu50, COL, 8325, MW2, and MSSA476), and was the most comprehensive *S. aureus* microarray chip available when this study was carried out. Five micrograms each of genomic DNA from the test strain and reference strain (MRSA252) was labeled with Cy3 and Cy5, respectively, and competitively hybridized on the microarray slide using methods as previously described. Slides were scanned and data were extracted by GenePix. The microarray data were loaded into GeneSpring (Agilent Technologies) for normalization and analysis. An arbitrary cutoff of 2-fold was used to identify genes that were specific to a single strain. Genes with a ratio greater than the upper cutoff (2.0) were deemed specific to the test strain, genes with a ratio less than the lower cutoff were considered specific to the reference strain, and genes with ratio between 0.5 and 2 were classified as present in both strains.

### RNA Extraction and Northern Blot

A 1∶10 diluted overnight culture was subcultured for 3 h at 37°C, harvested, and subjected to bacterial cell wall lysis using 10 µg of lysostaphin (Sigma) for 10 min at 37°C. Total RNA was isolated using the Trizol method [49]. Digoxygenin-labeled RNA probes were prepared by *in vitro* transcription with T7 RNA polymerase using PCR-generated fragments as templates. The PCR fragments were generated using chromosomal DNA of *S. aureus* COL. For northern blot analysis, 10 µg of total RNA (5 µg for *RNAIII* detection) were separated in a 1% agarose gel containing 20 mM morpholinepropanesulfonic acid (MOPS), 5 mM sodium acetate, 1 mM EDTA, and 1.85% formaldehyde at pH 7.0. The gel was blotted onto a nylon membrane with 20× SSPE (3 M NaCl, 0.2 M NaH_2_PO_4_, and 0.02 M EDTA pH 7.4) by using a vacuum blotter. After 4 h, RNA was fixed on the membrane by UV cross-linking for 1 min. The membrane was stained with methylene blue to visualize 16 and 23S rRNA bands to control for successful blotting and the existence of equal amounts of total RNA in each lane. Band density was digitalized using AlphaEaseFC software (Alpha Innotech Corp., CA, USA).

### Reverse Transcription and Real-time Quantitative PCR for the Detection of PSM-α Production

Purified RNA (1 µg) was used for reverse transcription (RT) by using the SuperScript III first-strand synthesis system (Invitrogen). This cDNA product was used for real-time quantitative PCR (real-time qPCR) according to the iQ SYBR Green Supermix (Bio-Rad Laboratories, CA, USA) manufacturer’s instructions, in which a 25 µL reaction mixture contained primers (200 nM in each). The primers used were as follows: PSM-α1-F (5′–ATGGGTATCATCGCTGGCATCATTA) and PSM-α 3-R (5′–ACCTAAAAATTTACCAAGTAAATCT) for amplifying the PSM-α transcript; 16S rRNA-F (5′–TCCTACGGGAGGCAGCAGT) and 16S rRNA-R (5′–GGACTACCAGGGTATCTAATCCTGTT) for a reference control. The reaction was performed in the iQ5 Multicolor real-time PCR detection system with iQ5 optical system software (Bio-Rad Laboratories, USA), and the reaction conditions were 95°C for 3 min, 1 cycle; 95°C for 10 s, 60°C for 30 s, 40 cycles; 95°C for 1 min, 50°C for 1 min, 1 cycle; then starting from 50°C for 10 s for melt curve with a 0.5°C increase after each cycle, 91 cycles.

### Statistics

The significances of strain differences in murine nasal colonization (Figure 2b), bacterial loads in each organ of mice infected with a non-lethal dosage of bacteria (Figure 4c), and the expression of various virulence factors or global regulators (Figure 7a–c) were determined using non-parametric Mann–Whitney tests. The statistical significance of differences in the adherence to A549 cells (Figure 2a) and the body weight changes of mice infected with non-lethal dosages of bacteria (Figure 4b) in different strains was determined using two-way analysis of variance (ANOVA) tests. Survivals of septic mice infected with lethal dosages of bacteria were compared between different strains by using a Log-rank (Mantel-Cox) test. The significances of differences between the different strains in virulence determinants were detected using a *t*-test. All data were analyzed using GraphPad Prism 5 for Windows (GraphPad Software, La Jolla, CA, USA).

## Supporting Information

Figure S1 The growth rates (a), biofilm productions (b, c) and antimicrobial susceptibility patterns (d) of Taiwan clone and Asian-Pacific clone of CA-MRSA ST59.
(TIF)

Table S1 Comparative genomics of Taiwan clone and Asian-Pacific clone of ST59 identified 3 genes and 28 genes respectively specific to Taiwan clone and Asian-Pacific clone.
(DOCX)

Table S2 Primers used in detecting various virulence factors of *S. aureus* (adapted from Infect Immun 2002;70∶631–641; Infect Immun 2002;70∶4987–4996; Lancet 2006;367∶731–739).
(DOCX)

Methods S1 (DOCX)
