# Supplementary material for: Characterization and Comparison of 2 Distinct Epidemic Community-Associated Methicillin-Resistant Staphylococcus aureus Clones of ST59 Lineage
Source: PLoS One. 2013 Sep 5;8(9):e63210. doi: 10.1371/journal.pone.0063210 (PMC3764004; doi:10.1371/journal.pone.0063210)
Supplement: Table S1 — Comparative genomics of Taiwan clone and Asian-Pacific clone of ST59 identified 3 genes and 28 genes respectively specific to Taiwan clone and Asian-Pacific clone. (DOCX) [file pone.0063210.s002.docx]

Table S1. Comparative genomics of Taiwan clone and Asian-Pacific clone of ST59 identified 3 genes and 28 genes respectively specific to Taiwan clone and Asian-Pacific clone.

| Common Name | Genbank | location | MRSA252 Product |
| --- | --- | --- | --- |
| Taiwan clone-specific ORFs | | |  |
| SAR1320 | 49483501 |  | hypothetical protein |
| SAR1317 | 49483498 |  | hypothetical protein |
| SAR1562 | 49483740 | prophage | phage integrase |
| Asian-pacific clone-specific ORFs | | |  |
| ccrA | 49482307 | SCC | site-specific recombinase |
| ccrB | 49482306 | SCC | site-specific recombinase |
| mecR1 | 49482292 | SCC | methicillin resistance protein MecR1 |
| SAR0025 | 49482278 | SCC | hypothetical protein |
| SAR0061 | 49482308 | SCC | putative membrane protein |
| SAR1860 | 49484022 | core genome | hypothetical protein |
| COL0424 | 21281729 | prophage |  |
| int | 49484247 | prophage | integrase |
| *sak* | 49484186 | prophage | staphylokinase precursor |
| SAR2040 | 49484187 | prophage | autolysin |
| SAR2041 | 49484188 | prophage | holin |
| *sep* | 15927524 | prophage | staphylococcal enterotoxin P precursor |
| SAR2044 | 49484191 | prophage | hypothetical protein |
| SAR2078 | 49482253 | prophage | hypothetical phage protein (pseudogene) |
| SAR2080 | 49484224 | prophage | hypothetical phage protein |
| SAR2082 | 48482253 | prophage | putative phage regulatory protein (pseudogene) |
| SAR2083 | 49484225 | prophage | putative single-strand DNA-binding protein |
| SAR2085 | 49484227 | prophage | hypothetical phage protein |
| SAR2086 | 49484228 | prophage | hypothetical phage protein |
| SAR2087 | 49484229 | prophage | hypothetical phage protein |
| SAR2088 | 49484230 | prophage | hypothetical phage protein |
| SAR2097 | 49484239 | prophage | hypothetical phage protein |
| SAR2098 | 49484240 | prophage | hypothetical phage protein |
| SAR2099 | 49484241 | prophage | DNA-binding protein |
| SAR2100 | 49484242 | prophage | putative repressor |
| SAR2101 | 49484243 | prophage | putative exonuclease |
| SAR2104 | 49484246 | prophage | putative lipoprotein |
| SAS0905 | 49484912 | prophage | DnaB-like helicase |
