# Supplementary material for: Characterization and Comparison of 2 Distinct Epidemic Community-Associated Methicillin-Resistant Staphylococcus aureus Clones of ST59 Lineage
Source: PLoS One. 2013 Sep 5;8(9):e63210. doi: 10.1371/journal.pone.0063210 (PMC3764004; doi:10.1371/journal.pone.0063210)
Supplement: Table S2 — Primers used in detecting various virulence factors of S. aureus (adapted from Infect Immun 2002;70∶631–641; Infect Immun 2002;70∶4987–4996; Lancet 2006;367∶731–739). (DOCX) [file pone.0063210.s003.docx]

Table S2. Primers used in detecting various virulence factors of *S. aureus* (adapted from Infect Immun 2002;70:631-641; Infect Immun 2002;70: 4987-4996; Lancet 2006;367:731-739).

| **PCR product** | **Primer** | **sequence (5’-3’)** | **Size of PCR product** |
| --- | --- | --- | --- |
| *sea* | SEA-1  SEA-2 | GAAAAAAGTCTGAATTGCAGGGAACA  CAAATAAATCGTAATTAACCGAAGGTTC | 560 |
| *seb* | SEB-1  SEB-2 | ATTCTATTAAGGACACTAAGTTAGGGA  ATCCCGTTTCATAAGGCGAGT | 404 |
| *sec* | SEC-1  SEC-2 | GTAAAGTTACAGGTGGCAAAACTTG  CATATCATACCAAAAAGTATTGCCGT | 297 |
| *sed* | SED-1  SED-2 | GAATTAAGTAGTACCGCGCTAAATAATATG  GCTGTATTTTTCCTCCGAGAGT | 492 |
| *see* | SEE-1  SEE-2 | CAAAGAAATGCTTTAAGCAATCTTAGGC  CACCTTACCGCCAAAGCTG | 482 |
| *seg* | SEG-1  SEG-2 | AATTATGTGAATGCTCAACCCGATC  AAACTTATATGGAACAAAAGGTACTAGTTC | 642 |
| *seh* | SEH-1  SEH-2 | CAATCACATCATATGCGAAAGCAG  CATCTACCCAAACATTAGCACC | 376 |
| *sei* | SEI-1  SEI-2 | CTCAAGGTGATATTGGTGTAGG  AAAAAACTTACAGGCAGTCCATCTC | 576 |
| *sej* | SEJ-1  SEJ-2 | TAACCTCAGACATATATACTTCTTTAACG  AGTATCATAAAGTTGATTGTTTTCATGCAG | 300 |
| *TSST-1* | TST-1  TST-2 | TTCACTATTTGTAAAAGTGTCAGACCCACT  TACTAATGAATTTTTTTATCGTAAGCCCTT | 180 |
| *ETA* | ETA-1  ETA-2 | ACTGTAGGAGCTAGTGCATTTGT  TGGATACTTTTGTCTATCTTTTTCATCAAC | 190 |
| *ETB* | ETB-1  ETB-2 | CAGATAAAGAGCTTTATACACACATTAC  AGTGAACTTATCTTTCTATTGAAAAACACTC | 612 |
| *PVL* | PVL-1  PVL-2 | ATCATTAGGTAAAATGTCTGGACATGATCCA  GCATCAASTGTATTGGATAGCAAAAGC | 433 |
| *fnbA* | FNBA-1  FNBA-2 | CACAACCAGCAAATATAG  CTGTGTGGTAATCAATGTC | 1362 |
| *icaA* | ICAA-1  ICAA-2 | GATTATGTAATGTGCTTGGA  ACTACTGCTGCGTTAATAAT | 770 |
| *sdrE* | SDRE-1  SDRE-2 | CAGTAAATGTGTCAAAAGA  TTGACTACCAGCTATATC | 767 |
| *cna* | CNA-1  CNA-2 | AGTGGTTACTAATACTG  CAGGATAGATTGGTTTA | ~560 |
| *hlg* | HLG-1  HLG-2 | GTCAYAGAGTCCATAATGCATTTAA  CACCAAATGTATAGCCTAAAGTG | 535 |
| *arcA* | ACME | GAGCCAGAAGTACGCGAG  CACGTAACTTGCTAGAACGAG |  |
