# Supplementary material for: Characterization and Comparison of 2 Distinct Epidemic Community-Associated Methicillin-Resistant Staphylococcus aureus Clones of ST59 Lineage
Source: PLoS One. 2013 Sep 5;8(9):e63210. doi: 10.1371/journal.pone.0063210 (PMC3764004; doi:10.1371/journal.pone.0063210)
Supplement: Methods S1 — (DOCX) [file pone.0063210.s004.docx]

Supplement methods

*Pulsed field gel electrophoresis*

Bacterial colonies grown overnight on blood agar plates were suspended in 10 mM Tris–0.1 mM EDTA and cast into gel plugs. The plugs were treated in lysis solution (6 mM Tris-HCl [pH 7.6], 1 M NaCl, 100 mM EDTA [pH 7.5], 0.5% Brij, 0.2% deoxycholate, 0.5% sodium lauroyl sarcosine, 30 􏰎g of RNase [DNase free] per ml, 1 mg of lysozyme per ml) with 1 mg of lysostaphin/ml at 37°C for 24 h and were further incubated in ESP buffer (0.5 M EDTA [pH 9 to 9.5], 1% sodium lauroyl sarcosine, 500 􏰎g of proteinase K per ml) at 50°C for 24 h. Plugs were thoroughly washed; then thin slices of the DNA plugs were cut and incubated overnight with 50 U of *SmaI* (New England Biolabs, Beverly, Mass.) at 25°C. Plugs were then loaded onto a 1% agarose gel, and PFGE was carried out with a CHEF Mapper XA system (Bio-Rad Labora- tories) at 14°C. An autoalgorithm mode was chosen, with the running molecular sizes ranging from 30 to 500 kb. The gel was stained with ethidium bromide and photographed with UV illumination.

*Biofilm formation assay*

The amount of biofilms formation in the wells was determined using a crystal violet staining method [1]. Briefly, 200-μl bacterial suspension of overnight culture was incubated in 96-well polystyrene microtiter plate at 37 °C for 24 hours. The suspension was removed and well was washed with PBS and stained with 1% crystal violet solution. After further wash for three times, the crystal violet was dissolved by 95% ethanol and detected at OD_595_. All isolates were tested in six replicates. We tested the biofilm forming capacity for all isolates under two different growth conditions; TSB supplemented with 0.5% glucose and TSB supplemented with 4% NaCl.

*Antibiotic susceptibility test*

The susceptibility of the MRSA isolates to 8 antibiotics, including mupirocin, chloramphenicol, ofloxacin, tetracycline, gentamicin, kanamycin, spectinomycin, erythromycin, fusidic acid, trimethoprim/sulfamethoxazole and rifampin, was determined by disc diffusion method according to the Clinical and Laboratory Standard Institute (2006) standards for antimicrobial susceptibility testing.

*Selection of clinical CA-MRSA strains for determination of virulence factors*

During 2003 and 2007, a collection of clinical CA-MRSA isolates from pediatric patients was stored at -80 °C in the strain bank of the research laboratory in Chang Gung Memorial Hospital. The isolates were labeled with consecutive numbers and the random integers were generated using the service provided by RANDOM.ORG (www.random.org). A subset of the first 82 isolates from the table of random number was selected for PCR-determining the carriage of specific virulence factors of *S. aureus*.

*Polymerase Chain Reaction (PCR) for SCCmec typing and screening of selected virulence determinants*

SCC*mec* typing of isolates was done using a multiplex PCR strategy described previously [2]. The control strains for SCC*mec* types I, II, III, and IVa, kindly provided by Keiichi Hiramatsu, were as follows: type I, NCTC10442; type II, N315; type III, 85/2082; and type IVa, JCSC4744. The strain TSGH-17, kindly provided by Chi- Chien Wang, was used as a control for SCC*mec* V_T_. The PCR screening of selected virulence determinants was performed in accordance with the method described elsewhere [3-5]. The primers used are listed in Table S2.

References

1. Cramton SE, Gerke C, Gotz F (2001) In vitro methods to study staphylococcal biofilm formation. Methods Enzymol 336: 239-255.

2. Huang YC, Hwang KP, Chen PY, Chen CJ, Lin TY (2007) Prevalence of methicillin-resistant Staphylococcus aureus nasal colonization among Taiwanese children in 2005 and 2006. J Clin Microbiol 45: 3992-3995.

3. Jarraud S, Mougel C, Thioulouse J, Lina G, Meugnier H, et al. (2002) Relationships between Staphylococcus aureus genetic background, virulence factors, agr groups (alleles), and human disease. Infection & Immunity 70: 631-641.

4. Peacock SJ, Moore CE, Justice A, Kantzanou M, Story L, et al. (2002) Virulent combinations of adhesin and toxin genes in natural populations of Staphylococcus aureus. Infect Immun 70: 4987-4996.

5. Diep BA, Gill SR, Chang RF, Phan TH, Chen JH, et al. (2006) Complete genome sequence of USA300, an epidemic clone of community-acquired meticillin-resistant Staphylococcus aureus. Lancet 367: 731-739.
